# Motor band sign is specific for amyotrophic lateral sclerosis and corresponds to motor symptoms

**DOI:** 10.1002/acn3.52066

**Published:** 2024-04-22

**Authors:** Charlotte Zejlon, Stefan Sennfält, Johannes Finnsson, Bryan Connolly, Sven Petersson, Tobias Granberg, Caroline Ingre

**Affiliations:** ^1^ Department of Neuroradiology Karolinska University Hospital Stockholm Sweden; ^2^ Department of Clinical Neuroscience Karolinska Institutet Stockholm Sweden; ^3^ Department of Neurology Karolinska University Hospital Stockholm Sweden; ^4^ Department of Medical Radiation Physics and Nuclear Medicine Karolinska University Hospital Stockholm Sweden

## Abstract

**Objective:**

Magnetic resonance imaging can detect neurodegenerative iron accumulation in the motor cortex, called the motor band sign. This study aims to evaluate its sensitivity/specificity and correlations to symptomatology, biomarkers, and clinical outcome in amyotrophic lateral sclerosis.

**Methods:**

This prospective study consecutively enrolled 114 persons with amyotrophic lateral sclerosis and 79 mimics referred to Karolinska University Hospital, and also 31 healthy controls. All underwent 3‐Tesla brain susceptibility‐weighted imaging. Three raters independently assessed motor cortex susceptibility with total and regional motor band scores. Survival was evaluated at a median of 34.2 months after the imaging.

**Results:**

The motor band sign identified amyotrophic lateral sclerosis with a sensitivity of 59.6% and a specificity of 91.1% versus mimics and 96.8% versus controls. Higher motor band scores were more common with genetic risk factors (*p* = 0.032), especially with *C9orf72* mutation, and were associated with higher neurofilament light levels (std. *β* 0.22, *p* = 0.019). Regional scores correlated strongly with focal symptoms (medial region vs. gross motor dysfunction, std. *β* −0.64, *p* = 0.001; intermediate region vs. fine motor dysfunction, std. *β* −0.51, *p* = 0.031; lateral region vs. bulbar symptoms std. *β* −0.71, *p* < 0.001). There were no associations with cognition, progression rate, or survival.

**Interpretation:**

In a real‐life clinical setting, the motor band sign has high specificity but relatively low sensitivity for identifying amyotrophic lateral sclerosis. Associations with genetic risk factors, neurofilament levels and somatotopic correspondence to focal motor weakness suggest that the motor band sign could be a suitable biomarker for diagnostics and clinical trials in amyotrophic lateral sclerosis.

## Introduction

Amyotrophic lateral sclerosis (ALS) is a clinically heterogeneous disease, typically presenting with focal extremity weakness or difficulty speaking and swallowing.^1^ The disease commonly generalizes to widespread motor dysfunction, resulting in death within 2–5 years after symptom onset. In addition, almost 50% of patients develop cognitive impairment and up to 15% meet the criteria for frontotemporal dementia.^2^ There is often a relatively long diagnostic delay in ALS, frequently a year or longer,^3^ partly due to a lack of reliable diagnostic biomarkers. The diagnosis is largely based on clinical findings, guided by diagnostic criteria such as the El Escorial criteria or the newer and simpler Gold Coast criteria.^4^, ^5^ In ALS, there is simultaneous involvement of upper (cortical) motor neurons (UMN) and lower (spinal/brainstem) motor neurons (LMN) and evidence of both is a prerequisite for diagnosis. In addition to the clinical examination, there is currently no reliable routine method of identifying UMN dysfunction. However, there is evidence suggesting that MRI might be used to this end, for example, by assessing focal brain iron deposition.^6^

Iron deposition is a common finding with aging,^7^ but is often increased in neurodegenerative diseases including ALS, where it is mainly found in the primary motor cortex.^6^ Several imaging studies (including 3‐ and 7‐Tesla MRI) have indicated that this accumulation of iron occurs preferentially in the cortical regions associated with the body area of symptom onset.^8^, ^9^ Postmortem studies have shown iron accumulation within microglia in the deep layers of the primary motor cortex in ALS patients and is thought to be associated with neuroinflammation.^10^, ^11^, ^12^ Microglia are considered important in the neuroinflammatory processes in ALS.^13^

These degenerative iron accumulations are observed as hypointensities in the primary motor cortex, also called the motor band sign (MBS), on T2‐, T2*‐ or susceptibility‐weighted imaging (SWI).^14^, ^15^ SWI is a gradient echo MRI sequence that uses both magnitude and phase data to improve the contrast from local susceptibility changes in the brain tissue, which improves the sensitivity in detecting iron content.^16^ Assessment of MBS has previously shown high sensitivity and specificity compared to healthy volunteers,^17^ but prospective validation in a clinical setting with consecutive patients undergoing investigations for possible ALS is lacking and precludes its use as a standard diagnostic procedure. Furthermore, its correlation to clinical status, including focal weakness, outcomes, biomarkers, and genetics is seldom described in detail. This paper therefore aimed to (1) evaluate a robust and clinically applicable measurement method for MBS; and (2) apply the method in a population cohort of newly diagnosed ALS patients, ALS mimics and healthy controls to explore its sensitivity, specificity, and correlation to focal motor weakness as well as clinical outcomes, neurofilament light (NfL), and risk gene carriership.

## Subjects and Methods

### Participants

This prospective study consecutively enrolled 114 incident ALS patients diagnosed with probable, possible, or definite ALS according to the El Escorial criteria,^5^ at the ALS Clinical Research Centre at the Karolinska University Hospital, Stockholm, Sweden. The patients underwent brain MRI according to a standardized protocol on a single MRI scanner between April 2019 and February 2023. Spouses of the ALS patients with no history of neurological disease were also offered inclusion in the study as healthy controls (*n* = 31). Retrospectively, all patients referred to the clinic under suspicion of ALS, investigated on the same scanner during the same period but ultimately diagnosed with a different condition were included as an ALS mimic group (*n* = 79). The most common final diagnoses in this group were spinal stenosis (11.4%), benign fasciculations (10.1%), Parkinson's disease (10.1%), and polyneuropathy (10.1%) (Table S1).

### Clinical and biochemical procedures

Clinical data were obtained retrospectively from medical records and from the Swedish Motor Neuron Disease Quality Registry,^18^ including complete information for patient characteristics such as age, sex, site of onset, and date of onset.

Physical impairment in ALS patients was assessed using the revised ALS Functional Rating Scale (ALSFRS‐R). The scale is composed of 12 items measuring impairment in several domains: bulbar items 1–3; fine motor items 4–6; gross motor items 7–9; and respiratory items 10–12.^19^ The gross motor items predominantly evaluate lower limb/truncal movements whereas fine motor items reflect hand/finger movements. Each item has a maximum score of four, a lower score indicating impaired function, yielding a maximum score of 12 for each sub‐score and 48 in total. ALSFRS‐R was available for all ALS patients and assessed within a median of 0.7 months (interquartile range [IQR] 1.3) of the MRI. Progression rate was calculated by subtracting the ALSFRS‐R score from 48 and dividing the resulting number by the time (in months) since diagnosis, thus yielding the decline in ALSFRS‐R per month.

A total UMN score was calculated by assessing the presence of UMN signs in each extremity and bulbar region, respectively, yielding a score of 0–5. UMN signs were defined as spasticity, hyperreflexia (in relation to weakness), a plantar extensor response, or a positive Hoffmann sign.

Cognitive impairment in ALS patients was assessed using the Edinburgh Cognitive and Behavioural ALS Screen (ECAS), which evaluates cognition and behavioral changes specific for ALS.^20^ ECAS was available for 84 (73.7%) of ALS patients and the assessment was performed within a median of 1.6 months (IQR 1.6) of the MRI.

NfL is increased in cerebrospinal fluid as a result of neuronal injury and is commonly used as a diagnostic and prognostic biomarker in ALS.^21^ NfL was available for 113 (99.1%) of ALS patients and was obtained within a median of 0.1 months (IQR 1.4) of the MRI.

Genetic testing was performed in 98 ALS patients (86.0%), including the most common pathogenic genetic mutations in ALS such as *SOD1, C9orf72, TBK1, TDP‐43, FUS*, and *OPTN*.

Survival was assessed at the end of December 2023, a median of 34.2 (IQR 22.7) months after the MRI.

### Neuroimaging procedures

Each participant underwent a full brain MRI protocol, including 3D T1‐weighted, 3D T2‐weighted, 3D T2‐FLAIR, and diffusion‐weighted imaging as well as 3D SWI. The scans were performed on a Prisma^Fit^ 3‐Tesla MRI scanner (Siemens Medical Systems, Erlangen, Germany) at the Department of Neuroradiology, Karolinska University Hospital, using a 64‐channel head coil. The parameters of the 3D SWI sequence were as follows: repetition time 28 ms, echo time 20 ms; field of view 190 × 220 mm; voxel size 0.9 × 0.9 × 2.0 mm; bandwidth 120 Hz/pixel; flip angle 15° and an acquisition time ~ 4:30 min.

**Figure 1:**
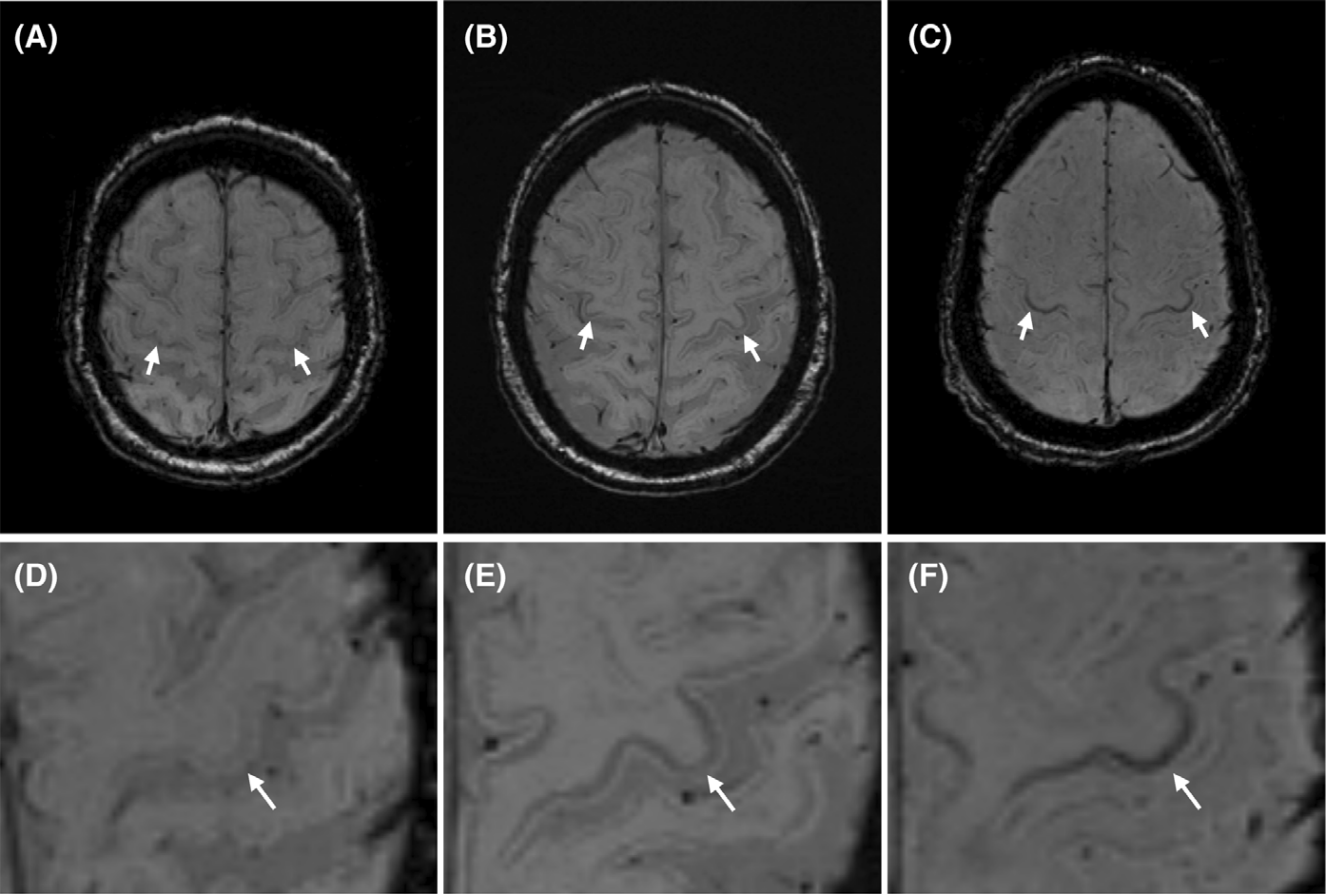
Motor band sign grading. Different grades (0–2) of primary motor cortex hypointensities, the motor band sign, on axial susceptibility‐weighted brain MRI in the intermediate region. D–F showing magnifications of A–C, respectively. A 74‐year‐old male with isointense (Grade 0) motor cortex (D); a 73‐year‐old male with mildly hypointense (Grade 1) motor cortex (B and E), and a 70‐year‐old male with marked hypointense (Grade 2) motor cortex (C and F).

**Figure 2:**
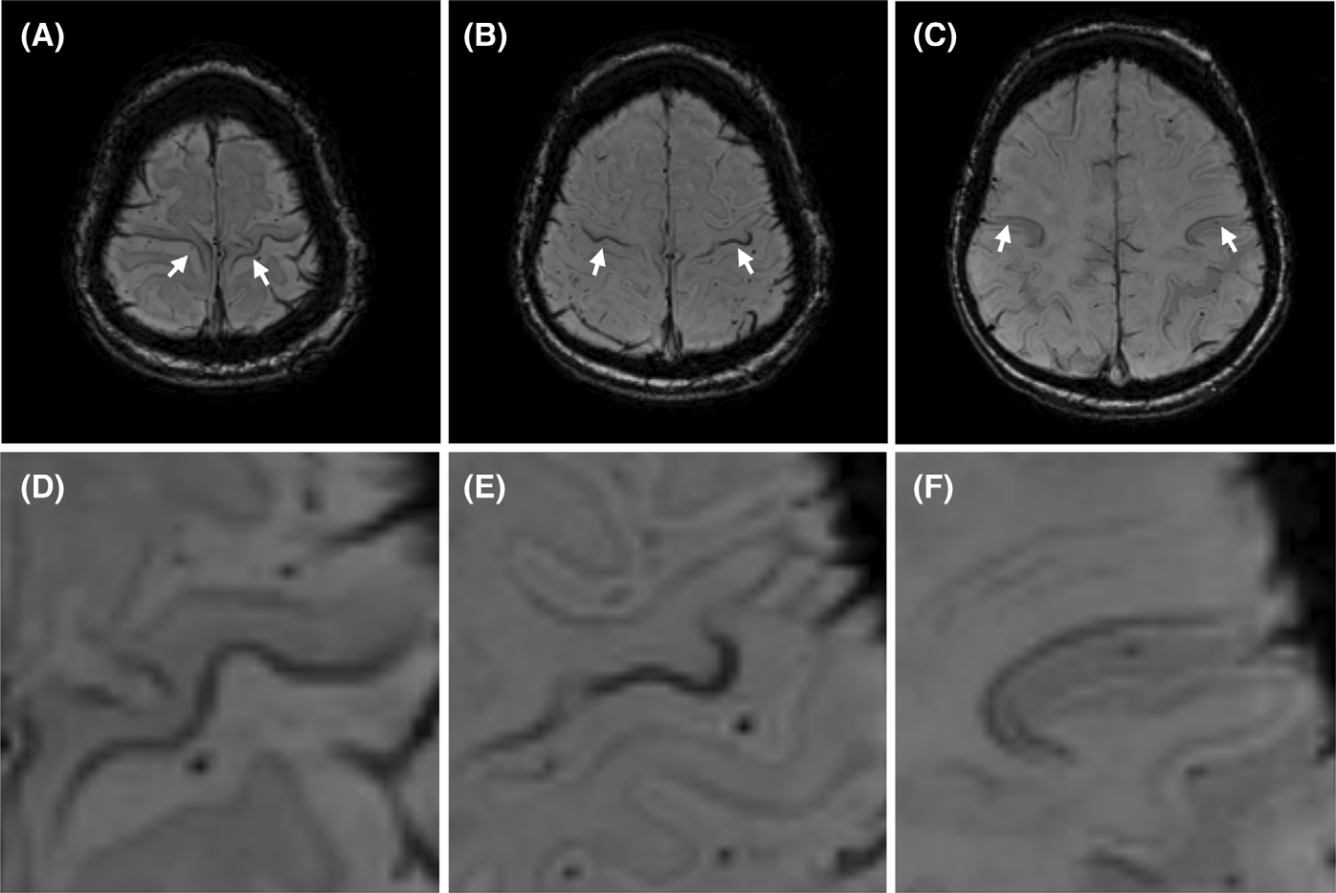
Motor band sign topographical classification. Anatomical distribution of primary motor cortex hypointensities, the motor band sign, on axial susceptibility‐weighted brain MRI in a 72‐year‐old female patient with dysarthria and mild motor weakness in the upper and lower extremities. D–F showing magnifications of A–C, respectively. There was marked hypointensities in the medial (A and D), intermediate (B and E), and lateral (C and F) regions of the motor cortex.

All SWI sequences were assessed on radiological PACS workstations (IDS 7, Sectra AB, Stockholm, Sweden) independently by a senior consultant neuroradiologist (J.F.), a neuroradiology fellow (B.C.) and a radiology resident (C.Z.). All raters were blinded to the participant's age, sex, their clinical diagnosis, and other clinical information. The raters evaluated the degree of hypointensity in the motor cortex on SWI using a three‐point ordinal scale from 0 to 2, as previously reported^17^: isointense (0), mildly hypointense (1), and markedly hypointense (2) compared to surrounding gyri (Fig. 1). The human primary motor cortex is somatotopically organized: the most medial parts are associated with motor function in the lower extremities, the most lateral parts with motor function in the face and tongue, whereas the intermediate region is associated with motor function of the upper extremities.^22^ Therefore, the degree of hypointensity was scored individually for these three regions of each hemisphere (Fig. 2), yielding a total MBS score of 0–12. Hypointensities spanning several regions received a score for each region. The gross motor items of the ALSFRS‐R predominantly evaluate lower extremity/truncal movements represented in the medial part of the homunculus, the fine motor items reflect hand/finger movements represented in the intermediate region, whereas the bulbar items/symptoms are represented in the lateral region.

The ratings were performed once by the two junior raters and twice by the senior rater (J.F). The first set of ratings by the senior rater (J.F.) were used for all analyses. The two sets of ratings by J.F. were used to calculate intrarater agreement and the ratings from all three raters were used to calculate interrater agreement.

### Statistical analysis

Categorical variables, including genetics, were summarized as proportions (percent) and the chi‐squared test was used to assess group differences. Continuous variables were reported as median (IQR) or mean ± standard deviation (SD). Group differences were assessed using the Mann–Whitney *U*‐test for two comparator groups, or the Kruskal–Wallis test for ≥3 comparator groups.

A weighted Fleiss' kappa was performed to assess inter‐rater reliability between the three raters and a weighted Cohen's kappa was used for the intra‐rater reliability measures.^23^, ^24^ Values greater than 0.6 were considered good reliability.

The correlation between hypointensities in the different areas of the primary motor cortex (ordinal scale, 0–2) was assessed using the Spearman's correlation coefficient. This test was also used to assess the correlation between laterality of MBS and motor symptoms. Laterality of motor symptoms were assessed on a scale of −2 to 2 (right predominant to left predominant) and MBS laterality was calculated by taking the total right hemisphere MBS score minus that of the left (−6 to 6, left to right). These two laterality scores were then analyzed for correlation. All correlation analyses only included ALS patients with MBS (*n* = 68).

Linear regression analyses were used to assess the association between regional MBS scores and clinical symptoms. We constructed separate models for each combination of primary motor cortex regional MBS score (both hemispheres added together) and ALSFRS‐R sub‐score and ECAS, adjusted for age, sex, and the total MBS score. Linear regression analyses were also performed to assess the association between the total MBS score and the total ALSFRS‐R, as well as levels of NfL and the total UMN score, adjusted for age and sex (and for UMN also for total ALSFRS‐R). Results were presented as the standardized beta (std. *β*).

A Cox regression model was constructed to assess the association between the total MBS score and survival. The model was adjusted for age, sex, and the total ALSFRS‐R.

For all analyses, a *p*‐value <0.05 was pre‐defined as statistically significant, except for the Spearman's correlation coefficient where a *p*‐value of <0.01 was used.

## Results

### Participant characteristics

Among the 114 ALS patients, the mean age at diagnosis was 64.0 ± 12.5 years and 64 (56.1%) were males. (Table 1). This compares to 62.5 ± 15.8 years and 46 males (58.3%) among the ALS mimics and 63 ± 14.3 years and 10 males (32.3%) among the healthy controls. There were 35 (29.9%) ALS patients with bulbar onset, 43 (36.8%) with upper extremity onset, 34 (29.1%) with lower extremity onset, while there were no patients with respiratory onset.

**Table 1:** Characteristics in the ALS patients, grouped by total motor band sign score.

	Total *N* = 114	Total MBS score			
		0 *n* = 46 (40.4%)	1–3 *n* = 27 (23.7%)	4–5 *n* = 24 (21.1%)	≥6 *n* = 17 (14.9%)
Age at MRI, median (IQR), mean ± SD	65 (16), 64.0 ± 12.5	65 (18), 63.5 ± 14.0	67 (13) 67.7 ± 8.4	64 (16), 64.0 ± 9.9	62 (24), 59.8 ± 15.6
Sex (male), *n* (%)	64 (56.1)	32 (69.6)	11 (39.3)	13 (54.2)	9 (52.9)
Months from onset to MRI, median (IQR)	14.3 (14.1)	14.6 (16.5)	10.1 (8.0)	14.5 (16.2)	16.4 (12.3)
Genetics, *n* (%)**, ^a^					
Negative on sequencing	72 (73.5)	33 (76.7)	19 (82.6)	13 (76.7)	7 (50.0)
*ATXN8*	2 (2.0)	0	0	0	2 (14.3)
*C9orf72*	17 (17.3)	5 (11.6)	4 (17.4)	4 (22.2)	4 (28.6)
*OPTN*	1 (1.0)	1 (2.3)	0	0	0
*SOD1*	4 (4.1)	3 (7.0)	0	1 (5.6)	0
*TBK1*	2 (2.3)	1 (2.6)	0	0	1 (7.7)
Clinical measures					
Total ALSFRS‐R, median (IQR)	40 (7)	40 (8)	40 (6)	42 (7)	39 (12)
ALSFRS‐R sub‐scores, median (IQR)					
Bulbar	11 (3)	12 (2)	10 (5)	10 (3)	10 (5)
Fine motor	9 (3)	9 (2)	9 (4)	11 (3)	9 (3)
Gross motor	9 (5)	9 (5)	11 (3)	8 (4)	7 (5)
Respiratory	12 (1)	12 (1)	12 (1)	12 (1)	12 (4)
Progression rate, median (IQR)	0.47 (0.58)	0.37 (0.63)	0.75 (0.48)	0.42 (0.66)	0.63 (0.59)
Onset area					
Bulbar	34 (29.8)	8 (17.4)	11 (40.7)	10 (41.7)	5 (29.8)
Upper extremity	46 (40.4)	23 (50.0)	12 (44.4)	7 (29.2)	4 (23.5)
Lower extremity	34 (29.8)	15 (32.6)	4 (14.8)	7 (29.2)	8 (47.1)
Biochemical markers					
Neurofilament light, median (IQR)*, ^b^	5280 (5437)	4763 (4327)	5280 (6146)	6890 (8700)	5610 (6233)

IQR, interquartile range; MBS, motor band sign; SD, standard deviation.

^a^ 16 patients missing.

^b^ 1 patient missing.

* *p* < 0.05;

** *p* < 0.01.

To assess representativeness, we compared the 114 ALS patients in the present study cohort to a larger prospective cohort including 353 ALS patients diagnosed between 2016 and 2021 in Stockholm, who were deemed as representative of all ALS patients in the source population (Table S2). Site of onset differed, with a higher proportion of patients with bulbar and respiratory onset in the comparison cohort, but age and sex distribution were similar.

### MBS in ALS patients, ALS mimics, and controls

**Figure 3:**
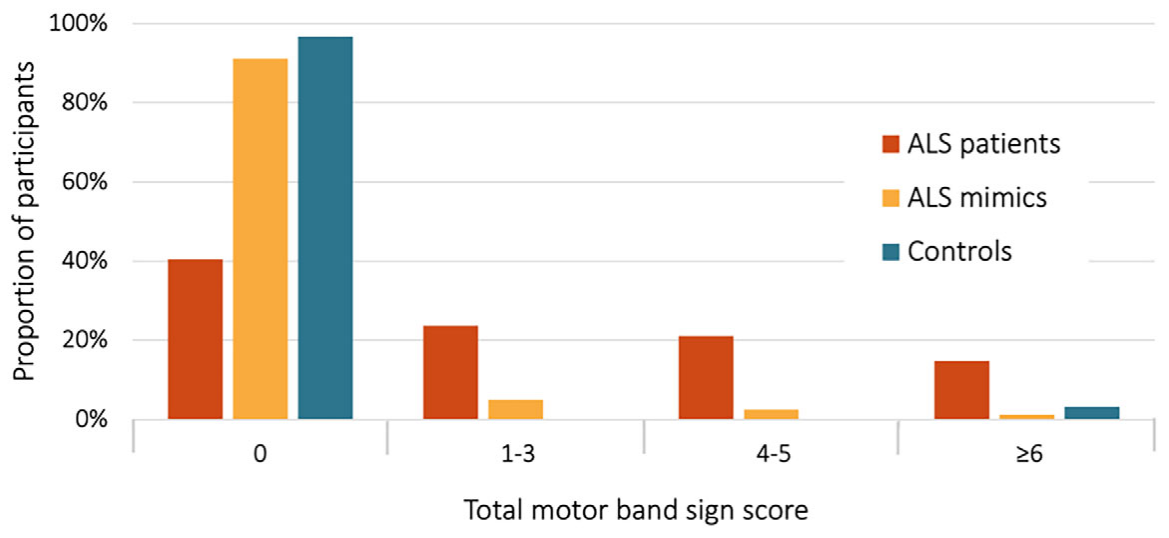
Distribution of the total motor band sign score in 114 ALS patients, 79 ALS mimics, and 31 controls.

The median time from symptom onset to MRI was 14.3 (IQR 14.1) months among the ALS patients. The MBS (a minimum score of one) was seen in 68 ALS patients (59.6%) compared to seven in the ALS mimics group (8.9%), and in one of the controls (3.2%), *p* < 0.001 for both comparisons. For a cutoff set at an MBS score of one (presence of hypointensities in any region of the primary motor cortex on either side) our results translate to a sensitivity of 59.6% and a specificity of 91.1% versus mimics and a specificity of 96.8% versus controls (Fig. 3).

The ALS patients were divided into four groups based on the total MBS score: 0 (*n* = 46 [40.4%]), 1–3 (*n* = 27 [23.7%]), 4–5 (*n* = 24 [21.1%]), ≥6 (*n* = 17 [14.9%]) (Table 1). This grouping was done arbitrarily to produce roughly equal group sizes. There were no statistically significant group differences with respect to age, sex, median ALSFRS‐R, median time from symptom onset to MRI, or site of onset. However, the MBS tended to be more prevalent in bulbar‐onset patients relative to spinal onset patients, that is, those with motor symptoms from the extremities (77.1% vs. 51.2%, *p* = 0.04). Among the 98 patients who were screened for pathogenic genetic mutations, 26 (26.5%) were positive, 17 of these patients (17.3%) had the *C9orf72* mutation. The prevalence of mutations differed significantly between groups. In the group with the highest MBS score, 50.0% were positive for a pathogenic mutation compared to 22.6% in the patients from the other groups combined (*p* = 0.032). Notably, the prevalence of the *C9orf72* mutation increased substantially with higher MBS scores, increasing from 11.6% to 28% in those with an MBS of 0 and ≥6, respectively. Lastly, regression analysis revealed an association between higher levels of NfL and a higher MBS score (std. *β* 0.22, *p* = 0.019).

### Location of MBS in ALS patients

**Figure 4:**
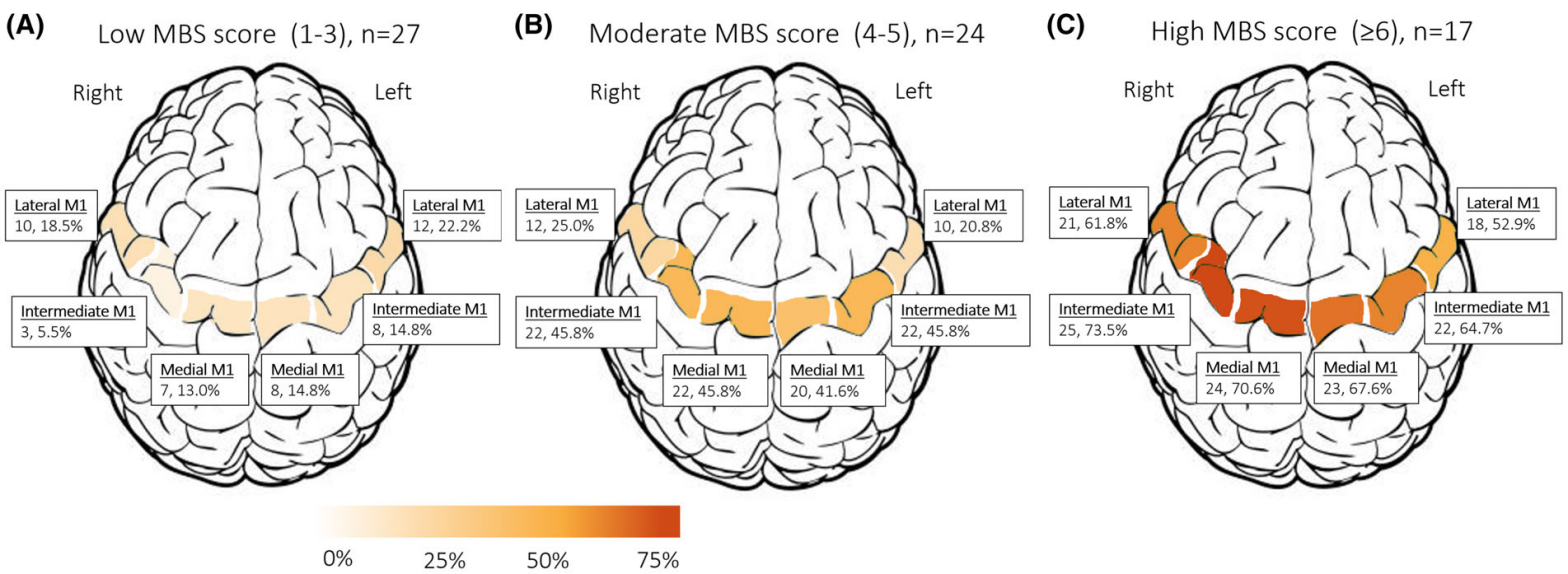
Regional motor band sign (MBS) score distribution by total score subgroup. Illustrations of the distribution in three different regions of the primary motor cortex (M1) (lateral, intermediate, and medial) shown separately for patients with low (1–3, A), moderate (4–5, B), and high (≥6, C) total MBS scores. The distribution is reported on group level: total regional MBS scores and the proportion of the maximum regional score (calculated as two times the number of patients as each region is rated on a scale of 0–2).

In the group with the lowest MBS score (1–3), the hypointensities were more commonly found in the lateral primary motor cortex, whereas in patients with a higher MBS score they were more prevalent in the intermediate and medial regions (Fig. 4). In all patients, the amount of hypointensities in the intermediate and medial regions were correlated to each other, both ipsilaterally and contralaterally (Table S3). Conversely, hypointensities in the lateral primary motor cortex were only correlated to the contralateral lateral region (except a significant correlation between the intermediate and the lateral region on the right side).

### Correlation between MBS and clinical symptoms

Upon assessment of survival (at a median of 34.2 months [IQR 22.7] from MRI), 67 out of 114 (58.8%) ALS patients had died, a median of 16.1 months (IQR 13.6) after the MRI.

Regression models including individuals with MBS, adjusted for age and sex, showed no significant association between the total MBS score and the total ALSFRS‐R (*p* = 0.11), progression rate (*p* = 0.21), ECAS (*p* = 0.11), total UMN score (*p* = 0.40), or survival (*p* = 0.27) (for the latter two, adjustments were also made for the total ALSFRS‐R). However, there was a strong association between regional MBS scores and focal motor weakness (ALSFRS‐R sub‐score), adjusting for the total MBS score (Table 2). Specifically, a negative association between medial region MBS scores and gross motor function (std. *β* −0.64, *p* = 0.001), between intermediate region MBS scores and fine motor function (std. *β* −0.51, *p* = 0.031), and between lateral region MBS scores and bulbar function (std. *β* −0.71, *p* < 0.001). Note that there was a significant positive association between regional MBS score and ALSFRS‐R sub‐score (i.e., indicating less focal weakness) for several regions, for example, the medial region and bulbar weakness. Additionally, there was a significant correlation between lateralization of MBS scores and lateralization of motor weakness (spearman's rho 0.31, *p* = 0.014). There was no association between regional MBS scores and respiratory function or ECAS.

**Table 2:** Linear regression models assessing the association between total/regional (including medial, intermediate, and lateral areas bilaterally) MBS scores and global/focal motor weakness (as indicated by ALSFRS‐R and its sub‐scores).

	ALSFRS‐R sub‐score				Total ALSFRS‐R
	Bulbar	Fine motor	Gross motor	Respiratory	
Total MBS score, adjusted for age and sex	−0.20 0.32	0.06 0.55	−0.22 0.021	0.06 0.57	−0.16 0.11
Regional MBS score, adjusted for age, sex and total MBS score					
Medial	0.66 <0.001	0.03 0.87	−0.64 0.001	0.20 0.34	
Intermediate	0.76 0.001	−0.51 0.031	−0.10 0.66	−0.22 0.36	
Lateral	−0.71 <0.001	0.20 0.18	0.42 0.003	−0.23 0.88	

Adjusted for total MBS score, age, and sex as indicated. All models only include ALS patients with MBS, *n* = 68, and show results as standardized beta coefficients and *p*‐values (the latter below the former). Highlighted in gray are results predictive of functional impairment (negative association since a lower ALSFRS‐R score indicates dysfunction), significant at the *p* < 0.05 level.

ALRFRS‐R, revised ALS Functional Rating Scale; MBS, motor band sign.

### Intra‐ and inter‐rater reliability of motor band sign assessment

There was good inter‐rater (*κ* = 0.632, *p* ≤ 0.001) and intra‐rater (*κ* = 0.668, *p* ≤ 0.001) agreement.

## Discussion

In this study, we evaluated a simple method for assessing and quantifying neurodegenerative susceptibility changes in the primary motor cortex, that is, the motor band sign (MBS), and demonstrated its clinical relevance as a biomarker for ALS in 114 ALS patients, and also in 79 ALS mimics, and 31 healthy controls.

Previous work on the MBS has mainly been performed at lower field strength (1.5‐Tesla), with traditional T2*‐weighted imaging, in smaller populations, or focusing mainly on distinguishing ALS patients from controls. In a recent meta‐analysis, we found that the MBS was more prevalent in ALS compared to controls with an odds ratio of 10.85 (95% CI: 3.74–31.44).^6^ Importantly, we also found a large variability in the sensitivity and specificity for differentiating ALS patients from controls, ranging from 24% to 100% and from 45% to 98%, respectively. These large variations in the previous literature motivated us to investigate prospectively, in a consecutive clinical population, how MBS fared as an imaging biomarker based on clinical state‐of‐the art 3‐Tesla brain MRI with SWI. To increase the clinical utility of our investigations we included a large group of ALS mimics in addition to healthy controls and assessed the topographical correlation to focal motor symptoms.

We found a limited sensitivity of MBS in identifying ALS (59.6%), but a high specificity both when compared to ALS mimics (91.1%) and healthy controls (96.8%). This is largely in line with previous literature showing a higher specificity than sensitivity.^6^ Of note, however, is that we found a substantially lower sensitivity compared to a recent smaller study from Japan using a methodology similar to ours, reporting a sensitivity and specificity of 92% and 100%, respectively, as compared to controls.^17^ This difference was not explained by variation in patient characteristics: disease duration was 14.3 months in our study compared to 10 months in the Japanese study, mean age was 64 years compared to 66 years, and ALSFRS‐R was 40 compared to 43. Also, the Japanese study used a higher MBS score cutoff of three, which would be expected to lower sensitivity. This suggests that the method needs to be replicated in larger and diverse patient cohorts for standardization and to further establish the expected distribution of MBS.

Nevertheless, our results highlight the potential of MBS in a real clinical setting in distinguishing ALS from other neurodegenerative conditions, which is also supported by previous research.^25^ This is an important aspect, as accurate and early diagnosis of ALS remains challenging due to the heterogeneity in clinical presentation and the absence of a definitive diagnostic test. Notably, the ALS mimics group consisted of a clinically highly relevant comparative group with 79 consecutive patients initially referred to the clinic on suspicion of ALS, but finally diagnosed with other (primarily neurological) conditions. They showed a higher degree of MBS than the healthy controls, suggesting that although relatively specific for ALS, primary motor cortex iron accumulation can also occur in other neurological disorders. Whether the accumulations are more often seen in brain regions other than the motor cortex remains to be determined. One control had a total MBS score of 6, with one point per region. The hypointensity was subtle and evenly distributed throughout the motor cortex. This subject is a healthy woman in her 60s with no history of a neurological disease or neurological symptoms.

Interestingly, the topographic location of MBS in the motor cortex was strongly associated with focal motor weakness in ALS patients, reflecting the somatotopic organization of the primary motor cortex.^22^ In ALS patients, while adjusting for total MBS, medial region MBS was strongly associated with gross motor dysfunction, intermediate region MBS with fine motor dysfunction, and lateral region MBS with bulbar symptoms and there was also a correlation between lateralization of MBS and lateralization of motor symptoms. Notably, those with relatively higher MBS scores in specific regions of the motor cortex, were less likely to experience weakness in non‐corresponding parts of the body. For example, those with a higher burden of MBS in the medial regions were less likely to show bulbar dysfunction. We interpret this as supportive of the somatotopic correlation. The topographic correlation to clinical symptoms has previously been shown by others, for example, grade of atrophy or MBS, in relation to site of onset.^8^, ^9^, ^26^ The relationship implies that MBS, as a radiological marker, could reflect the clinical manifestations of ALS, corroborating the pathophysiological relevance of the findings.

Of note however, is the lack of association between MBS and UMN signs, presumably a marker of cortical pathology. However, our scoring of UMN signs was retrospective, rudimentary, and did not include a dedicated scoring procedure nor a validated scale. The signs might not have been appreciated by the examiner, properly documented, or may have been masked by profound lower motor dysfunction.

Moreover, our data revealed no correlation between MBS and cognitive impairment in ALS patients, suggesting that MBS might primarily reflect motor neuron pathology. Also, the lack of association between MBS and total ALSFRS‐R, survival, or disease progression rate suggests that MBS might not be a reliable prognostic biomarker in its current form. However, this has to be explored further since a correlation to progression rate has been reported by others.^17^

An intriguing observation was the higher prevalence of confirmed genetic mutations in ALS patients with higher MBS scores. This suggests a potential genetic influence on the development of MBS, further emphasizing the heterogeneity of ALS and the role of genetic factors in disease manifestations. Specifically, the prevalence of the *C9orf72* mutation in screened patients increased from 11.6% to 28% in those with an MBS of 0 and ≥6, respectively. This is particularly interesting since the *C9orf72* mutation is associated with dysfunctional, pro‐inflammatory microglia,^27^ which are thought to be important in the pathogenesis in ALS,^13^ and specifically for the cerebral iron accumulation that occurs in the disease.^10^, ^11^, ^12^ Neuropathological studies suggest that the MBS reflects iron accumulation in microglia in the deep layers of the motor cortex.^10^, ^11^, ^12^ However, it is unknown if this is directly causing neuronal injury or if it is a marker of a different underlying pathological process, associated with neuroinflammation. The observed clinical‐radiological correlation would suggest that iron accumulation is indeed correlated to focal neuronal injury, and we also observed a correlation of MBS score and levels of NfL. However, there was no association between the time from symptom onset and the amount of iron accumulation which might be expected if the accumulation was a cumulative process over the disease trajectory. More studies are needed to confirm the correlation between MBS and the *C9orf72* mutation as well as MBS and NfL.

Thus, many questions remain to be answered, for example, (1) whether iron accumulation is truly a marker of neuronal death, (2) at which stage in the disease process iron accumulation is initiated (the shortest time from onset to imaging in our MBS positive ALS patients was 3 months), and (3) if it is present in other parts of the brain. This warrants further research that could preferably be conducted using more sensitive imaging techniques (e.g., Quantitative Susceptibility Mapping,^28^ or ultra‐high field strength MRI) and larger patient cohorts. As these methods are noninvasive, they might also be applied to presymptomatic at‐risk individuals, exploring at which time they are first notable and subsequent temporal evolution. In this study, we evaluated the ability of MBS to differentiate ALS from non‐motor neuron disease mimics and healthy controls. Future studies should ideally also study its role in identifying/differentiating PLS and PMA.

### Generalizability

We compared our patients to those of a different study including a larger sample deemed as representative of all ALS patients in the source population. The two cohorts were similar in respect to age and sex but differed in the distribution of site of onset: notably a much smaller proportion of respiratory onset in the present study cohort. The reason for this is likely that the respiratory onset patients were unable to remain in the supine position for a prolonged period (as is necessary for an MRI scan) due to dyspnea and/or use of non‐invasive ventilation. It is unclear how this discrepancy might have influenced our results, but we did not observe any association between MBS and respiratory function.

### Limitations

This study has some limitations. Ideally, the healthy control group could have been larger but, in this study, we decided to enroll spouses of the ALS patients to match known and unknown potential demographical confounders as accurately as possible. Further, there was good inter‐rater and intra‐rater agreement. However, the proposed cutoffs for level of reliability for both the Cohen's and the Fleiss' kappa are debated and should only be viewed as a rough indication. Longitudinal assessments of MBS is planned under this study protocol but due to the disease progression, few participants are able to perform follow‐ups and thus more data collection is needed to have presentable results. Such assessment, however, could provide indications whether iron deposition in the primary motor cortex accumulates with time and whether MBS responds to therapies.

## Conclusions

Primary motor cortex susceptibility (MBS) has high specificity but relatively low sensitivity for identifying ALS in a consecutive clinical cohort, including ALS mimics. Moreover, it is more prevalent in patients with genetic mutations associated with the disease and is correlated to higher levels of NfL. Regional MBS scores are specifically associated with focal motor weakness, corresponding topographically to the somatotopic organization of the primary motor cortex, corroborating the pathophysiological relevance. Our findings suggest that the MBS may be a diagnostic imaging biomarker for ALS. Its predictive value and potential role for clinical trials and treatment monitoring remains to be determined.

## Conflicts of Interest

C.I. has consulted for Cytokinetics, Pfizer, BioArctic, Novartis, Tikomed, Ferrer, and Mitsubishi. She is also a DMC member for Appelis, a Pharmaceutical Board member of Tobii Dynavox and Stiching, and a Scientific Advisory council member for the international Alliance. There are no other conflicts of interest to declare.

## Author Contributions

Tobias Granberg and Caroline Ingre initiated the study. Caroline Ingre, Tobias Granberg, and Stefan Sennfält applied for ethical approvals. Tobias Granberg and Sven Petersson designed the MRI protocol. Charlotte Zejlon, Johannes Finnsson, and Bryan Connolly evaluated imaging data. Stefan Sennfält evaluated clinical data and performed statistical analyses. Charlotte Zejlon and Stefan Sennfält drafted the manuscript. All authors revised the manuscript critically. All authors approved the final version of the manuscript.

## Supporting information


Table S1.

## Data Availability

Data on patient characteristics and imaging can be obtained from the original data sources or from the authors with the appropriate approval from an ethical review board.
