# Supplementary material for: Motor band sign is specific for amyotrophic lateral sclerosis and corresponds to motor symptoms
Source: Ann Clin Transl Neurol. 2024 Apr 22;11(5):1280–9. doi: 10.1002/acn3.52066 (PMC11093233; doi:10.1002/acn3.52066)
Supplement: Supplementary file 1 — Table S1. [file ACN3-11-1280-s001.docx]

Supplementary material

*Motor band sign is specific for amyotrophic lateral sclerosis and corresponds to motor symptoms*

| **Supplementary table 1** Final diagnoses in ALS mimics. Frequency (% of total) | |
| --- | --- |
| Spinal stenosis | 9 (11.4) |
| Benign fasciculations | 8 (10.1) |
| Parkinsons disease | 8 (10.1) |
| Polyneuropathy | 8 (10.1) |
| Herniated disc | 3 (3.8) |
| MSA-P | 3 (3.8) |
| Multiple sclerosis | 3 (3.8) |
| FTD | 2 (2.5) |
| Myastenia gravis | 2 (2.5) |
| Hereditary spastic paraparesis | 2 (2.5) |
| Alzheimers disease | 1 (1.3) |
| Cavernoma | 1 (1.3) |
| Chiari type 1 | 1 (1.3) |
| Epilepsy | 1 (1.3) |
| Essential tremor | 1 (1.3) |
| Foraminal stenosis | 1 (1.3) |
| Ischemic stroke | 1 (1.3) |
| Monomelic amyotrophy | 1 (1.3) |
| Mononeuropathy | 1 (1.3) |
| Multifocal motor neuropathy | 1 (1.3) |
| Psoriasis arthritis | 1 (1.3) |
| Welander’s distal myopathy | 1 (1.3) |
| Not diagnosed | 19 (24.1) |
| Total | 79 |

| **Supplementary table 2** Patient characteristics in the study cohort versis in a comparison cohort from a different study  IQR=Interquartile range | | |
| --- | --- | --- |
|  | **Study cohort**  N=114 | **Comparison cohort**  N=353 |
| Age, median (IQR) | 65.0 (16) | 68.0 (14) |
| Sex (male), n (%) | 64 (56.1) | 177 (50.1) |
| Site of onset, n (%) * |  |  |
| Bulbar | 34 (29.8) | 136 (38.5) |
| Upper extremity | 46 (40.4) | 85 (24.1) |
| Lower extremity | 34 (29.8) | 117 (33.1) |
| Respiratory | 0 | 15 (4.2) |
| * Statistically significant difference between groups, p<0.05  § For the study cohort median age refers to the age at MRI whereas for the comparison cohort, median age refers to the age at the initial health care visit related to ALS.  IQR=Interquartile range | | |

| **Supplementary table 3** Correlation matrix for regional MBS score on the right and left side. Only including ALS patient with MBS, n=68. Spearman correlation coefficient. Significant correlations (p<0.01) highlighted in orange  MBS=motor band sign | | | | | | | |
| --- | --- | --- | --- | --- | --- | --- | --- |
|  |  | **Right** |  |  | **Left** |  |  |
|  |  | **Medial** | **Intermediate** | **Lateral** | **Medial** | **Intermediate** | **Lateral** |
| **Right** | **Medial** | 1 | 0.68 | 0.21 | 0.78 | 0.46 | 0.03 |
|  | **Intermediate** | 0.68 | 1 | 0.31 | 0.61 | 0.77 | 0.18 |
|  | **Lateral** | 0.21 | 0.31 | 1 | 0.05 | 0.08 | 0.61 |
| **Left** | **Medial** | 0.78 | 0.61 | 0.05 | 1 | 0.50 | -0.14 |
|  | **Intermediate** | 0.46 | 0.77 | 0.08 | 0.50 | 1 | 0.00 |
|  | **Lateral** | 0.03 | 0.18 | 0.61 | -0.14 | 0.00 | 1 |
